# Comparing the measurement properties of the EQ-5D-5 L, SF-6Dv2, QLU-C10D and FACT-8D among survivors of classical Hodgkin’s lymphoma

**DOI:** 10.1007/s10198-024-01730-x

**Published:** 2024-10-17

**Authors:** Richard Huan Xu, Zuyi Zhao, Tianxin Pan, Andrea Monteiro, Hongfei Gu, Dong Dong

**Affiliations:** 1 Department of Rehabilitation Sciences, Faculty of Health and Social Sciences, Hong Kong Polytechnic University, Hong Kong, China; 2 Melbourne School of Population and Global Health, The University of Melbourne, Melbourne, Australia; 3 Department of Pharmacy Practice, College of Pharmacy, University of Illinois at Chicago, Chicago, USA; 4 Hongmian Cancers and Rare Disorders Charity Foundation of Guangzhou, Guangzhou, China; 5 JC School of Public Health and Primary Care, Faculty of Medicine, The Chinese University of Hong Kong, Hong Kong, China

**Keywords:** EQ-5D, SF-6D, QLU-C10D, FACT-8D, Preference-based measure, Measurement properties, Oncology, Lymphoma, I10

## Abstract

**Objective:**

This study aimed to evaluate the measurement properties of EQ-5D-5 L, SF-6Dv2, QLU-C10D, and FACT-8D in survivors of Classical Hodgkin’s Lymphoma (CHL).

**Methods:**

A cross-sectional, web-based survey was conducted from May to August 2022 to collect data. Chinese value sets were used to estimate the utility scores for EQ-5D-5 L, SF-6Dv2, and QLU-C10D, while the Australian value set was used for FACT-8D. The measurement properties assessed included ceiling and floor effects, convergent validity (assessing associations between similar dimensions/utility scores using Spearman’s rank correlation and intraclass correlation coefficient), and known-group validity (measures could differentiate health-related quality of life (HRQoL) between risk groups).

**Results:**

A total of 534 CHL survivors participated in the survey and completed the questionnaire. All dimensions of EQ-5D-5 L, SF-6D (except for vitality), QLU-C10D, and FACT-8D showed ceiling effects, ranging from 18 to 91.6%. The EQ-5D-5 L demonstrated the higher ceiling effects compared to other measures, with 33% of patients reporting full health on this scale. All 30 pairs of associations between similar dimensions from the four measures were statistically significant, with correlation coefficients ranging from 0.29 to 0.77. Regarding utility scores, the EQ-5D-5 L utility score showed a stronger correlation with SF-6Dv2 than with the other two measures. Statistically significant correlations of utility scores between the four measures were observed. EQ-5D-5 L can significantly differentiate HRQoL among all known-groups, while SF-6Dv2, QLU-C10D, and FACT-8D showed a less strong discriminant ability.

**Conclusions:**

EQ-5D-5 L outperformed SF-6Dv2 in terms of agreement with cancer-specific PRMs and discriminant ability. However, SF-6Dv2 showed stronger associations with similar dimensions of QLU-C10D and FACT-8D, indicating high convergent validity. The generic PBMs are sensitive enough to measure HRQoL in survivors of CHL.

## Introduction

Classical Hodgkin’s lymphoma (CHL) is a type of cancer that affects the lymphatic system. It is one of the leading causes of cancer-related deaths in adults, and its incidence rates are increasing worldwide [1]. Although CHL can affect people of all ages, it is more common among those aged over 15 years. Approximately 10,900 and 1200 new cases of CHL are diagnosed annually in the United States (US) and the United Kingdom (UK), respectively [1]. In China, more than 8000 new cases of CHL are reported each year [2]. CHL is prevalent among people aged under 50 years, accounting for approximately one third of all cancer cases in this age group. Patients with CHL typically experience painless swelling of the lymph nodes in their neck or chest regions. They may also present with weight loss and fever. Common symptoms of CHL include night sweats, fatigue and unintentional weight loss.

In recent years, new treatments for CHL have been introduced. For example, brentuximab vedotin has been shown to substantially improve remission in children and adolescents with CHL [3]. In addition, targeted immunotherapy has revolutionized the treatment of CHL. For instance, chimeric antigen receptor T-cell therapy is being investigated in clinical trials as a potentially effective treatment option [4]. Economic evaluation plays a crucial role in assessing the cost-effectiveness and value of interventions for CHL because health-care decision-makers must understand the economic implications of different treatment options and allocate their resources efficiently. Furthermore, CHL is a rare type of lymphoma with unique histological, immunophenotypic and clinical features. Relying solely on traditional clinical measures may not fully capture the holistic impact of the disease on patients’ lives.

Health-related quality of life (HRQoL) is a multidimensional concept that captures the impact of a disease on patients’ overall health and well-being. It reflects the variations in the health status of patients with lymphoma who have different clinical conditions and socioeconomic characteristics at various stages of disease care (with regard to their diagnosis, treatment and prognosis) [5]. HRQoL can be assessed using preference-based measures (PBMs). These measures estimate a utility score, which serves as a quantitative representation of an individual’s overall well-being or quality of life. This score is derived from capturing various dimensions of health and personal preferences, such as physical functioning, mental health, social interactions, and pain levels. By combining these dimensions into a single numerical value, the utility score provides a comprehensive and comparable metric for use in health economics, clinical decision-making, and policy development. It allows stakeholders to evaluate the effectiveness of medical interventions, allocate resources efficiently, and prioritize healthcare initiatives based on their perceived value and impact on patients’ lives [6].

The EQ-5D-5 L and SF-6D are the two most widely used generic PBMs. The EQ-5D-5 L consists of five generic health domains, with each domain assessed using a single question answered by the patient using a 5-level response scale. This measure has been applied to various health conditions to support rational decision-making regarding resource allocation in the global health-care sector [7]. The original SF-6D (SF-6Dv1) was derived from the 36-item Short-Form Health Survey (SF-36) and includes six dimensions [8]. The most recent update, SF-6Dv2, has undergone significant revisions to address ambiguities in its dimension levels and to unify the inconsistently positive and negative wording in the SF-6Dv1 [9]. It is important to note that the measurement properties of these measures vary across different health conditions, although they are widely used in different populations. In the context of cancer research, a key concern is the limited sensitivity of these measures to capture the relevant health issues due to the restricted number and type of dimensions [10].

Condition-specific measures are increasingly used to assess HRQoL in cancer clinical trials and economic evaluation. One such measure is the Functional Assessment of Cancer Therapy – Eight Dimensions (FACT-8D) [11], which is derived from the cancer-specific HRQoL profile measure known as the Functional Assessment of Cancer Therapy – General (FACT-G) [12]. The FACT-G is widely used in oncology clinical trials, either as a stand-alone questionnaire or included within specific modules. Another cancer-specific measure is the European Organisation for Research and Treatment of Cancer (EORTC) Quality of Life Utility Core – 10 Dimensions (QLU-C10D) [13], which focuses on the 10 dimensions derived from the EORTC Core Quality of Life questionnaire (QLQ-C30), one of the most commonly used instruments for assessing HRQoL in patients with cancer.

Utility scores measured by different PBMs vary due to differences in their descriptive systems/constructs and valuation methods [14]. Currently, there is a lack of psychometric evidence comparing the performance of cancer-specific PBMs with that of generic PBMs in patients with CHL. Considering the widespread use of the QLQ-C30 and FACT-G in cancer studies, it is important to determine whether the performance of the two conditional PBMs—the QLU-C10D and FACT-8D—is appropriate compared with the well-established generic PBMs for economic evaluation. Evidence for the measurement properties of PBMs in patients with CHL can provide valuable information for the choice and use of these instruments to support clinical decision and economic evaluation for this population. Therefore, the objective of this study was to evaluate the measurement properties of the EQ-5D-5 L, SF-6Dv2, QLU-C10D and FACT-8D in survivors of CHL.

## Methods

### Participants and data source

The data used for this analysis were obtained from a nationwide cross-sectional online survey that aimed to understand the HRQoL and social and health status of patients with CHL in China from September to November 2022. The survey was conducted with the assistance of House086, one of the largest organizations serving patients with lymphoma and their families in China. All of the respondents were House086 members. The inclusion criteria were as follows: (1) age ≥ 18 years, (2) able to read and write in Chinese, (3) no cognitive problems and (4) able to provide informed consent. We recruited the participants via House086’s internal social media platform, which uses both WeChat and QQ. Registered members received the study information and expressed interest in participating in the survey. Eligible members were invited to join a specific online survey group. Subsequently, staff members from the research team and House086 informed the respondents about the study and administered the survey. All respondents provided their informed consent before joining the survey. This study was approved by the Survey and Behavioural Research Ethics Committee of the Chinese University of Hong Kong (Ref. No.: SBRE-21-0437).

### Measures

#### EQ-5D-5 L

The EQ-5D-5 L comprises two sections, where the first section is a health state classification system with five dimensions, namely, mobility (MO), self-care (SC), usual activities (UA), pain/discomfort (PD) and anxiety/depression (AD). Each dimension has five response levels that range from “no problem” to “extreme problems.” All health states described by the classification system can be summarized as utility scores ranging from 0 (death) to 1 (full health), and health states worse than death are indicated by negative scores. In this study, the EQ-5D-5 L utility scores were estimated using the Chinese value set [15], with utility values ranging from − 0.391 to 1.0. The second section is the Visual Analogue Scale (EQ VAS), the scores of which range from 0 (worst imaginable health) to 100 (best imaginable health) and represent individuals’ global assessment of health.

#### SF-6Dv2

The SF-6Dv2 is derived from 10 items of the SF-36. The health state classification system of the SF-6Dv2 comprises six dimensions, namely, physical functioning, role limitation, social functioning, pain, mental health and vitality. The pain dimension has six response levels, while all of the other dimensions have five response levels. The Chinese SF-6Dv2 value set with utility values ranging from − 0.277 to 1 [16] was used in this study to estimate the utility scores.

#### EORTC QLU-C10D

Derived from the EORTC QLQ-C30, the QLU-C10D has 10 selected items that combine four functional domains (namely, physical, role, social and emotional) and six symptoms (namely, pain, fatigue, sleep, appetite, nausea and bowel problems) from the QLQ-C30. Each QLU-C10D item has four response levels: “not at all” (level 1), “a little” (level 2), “quite a bit” (level 3) and “very much” (level 4). In our study, the QLU-C10D utility scores were estimated using a Hong Kong Chinese value set [17], with utility values ranging from − 0.169 to 1.

#### FACT-8D

The FACT-8D comprises eight dimensions, namely, pain, fatigue, nausea, sleep, work, support, sadness and worry, which are derived from nine FACT-G items. Each dimension in the FACT-8D has the following five response options: “not at all” (0), “a little bit” (1), “somewhat” (2), “quite a bit” (3) and “very much” (4). The FACT-8D encompasses more than 390,000 possible health states (58 = 390,625). However, there is currently no available Chinese value set for the FACT-8D. In this study, the Australian value set of FACT-8D, with utility scores ranging from − 0.54 to 1, was used [18].

### Data analysis

Descriptive analysis was used to describe the patients’ background characteristics and health statuses. We reported the profiles of EQ-5D-5 L, SF-6Dv2, QLU-C10D, and FACT-8D, including the mean, standard deviation, median, and range of the utility scores. We also examined the ceiling effect (percentage of highest possible scores) and floor effect (percentage of lowest possible scores) at both the dimension and utility score levels. More than 15% of the sample reporting the lowest or highest score was indicative of a floor or ceiling effect, respectively [19]. Convergent validity was assessed using hypothesis testing. Thirty pairs of correlations between the four measures were hypothesized. For example, we expected a moderate-to-strong correlation between mobility assessed using the EQ-5D-5 L and physical functioning assessed using the SF-6D, a moderate-to-strong correlation between anxiety/depression assessed using the EQ-5D and emotional functioning assessed using the QLU-C10D, and a moderate-to-strong correlation between nausea assessed using the QLU-C10D and FACT-8D. Spearman’s correlation coefficient (r) was used to assess the strength of the hypotheses (weak, *r* ≤ 0.3; moderate, 0.31 ≤ *r* < 0.5; or strong, *r* ≥ 0.5) [20]. In addition, the agreement between measures (EQ-5D-5 L vs. SF-6D, EQ-5D-5 L vs. QLU-C10D, EQ-5D-5 L vs. FACT-8D, SF-6D vs. QLU-C10D, SF-6D vs. FACT-8D and QLU-C10D vs. FACT-8D) was assessed based on the intra-class correlation coefficient (ICC, > 0.7, satisfactory) and Bland–Altman plots. A bootstrap method (resamples = 1,000) was used to calculate the robust 95% confidence interval of the coefficient. Known-group validity was examined using analyses of variance (ANOVA). Participants were invited to answer a series of multiple-choice questions developed from literature review and research team discussions. These questions provided information about participants’ self-care ability, use of assistive tools, caregiver status, treatment status, and cancer stage. We assessed known-group validity through hypothesis testing. For instance, we hypothesized that patients who don’t need a caregiver would report higher utility scores than those who do. F-statistics were calculated to assess the discriminant efficiency of the measures in differentiating between participants with different conditions (i.e., self-care ability, using assistive tools, with a caregiver, treatment status, and cancer stage). A Bonferroni adjusted alpha level was used.

## Results

### Participants’ background characteristics

The sample characteristics of the patients who participated in this survey are presented in Table 1. Among 534 patients, 48.5% were men, 59.7% were married and 45.3% were actively employed. The most commonly diagnosed cancer stage was IIA (33.1%), followed by stage IV, indicating a large number of late-stage cancer diagnoses. The patients’ ages ranged from 18 to 82 years, with an average age of 35.6 years. Additionally, a majority of the participants (71.9%) held a tertiary degree or higher.

**Table 1:** Participant’s background characteristics

	*N* (Mean, SD)	% (Range)
Sex		
Male	259	48.5
Female	275	51.5
Educational level		
Secondary or below	150	28.1
Tertiary or above	384	71.9
Marital status		
Married	319	59.7
Unmarried	215	40.3
Employment status		
Active	242	45.3
Inactive	292	54.7
Caregiver		
No	256	47.9
Yes	278	52.1
Cancer stage		
I	20	3.7
IIA	177	33.1
IIB	68	12.7
III	81	15.2
IV	162	30.3
Unclear	26	4.9
Treatment status		
Being treated	281	52.6
Treatment completed	252	47.2
Age	35.6 (11.3)	18 ~ 82

### Measure’s profile and ceiling and floor effects

Tables 2 and 3 present the distribution of responses for the four measures. The mean (standard deviation) values of the EQ-5D-5 L utility and EQ-VAS scores were 0.89 (0.16) and 78.4 (17.4), respectively. Likewise, the mean utility scores for the SF-6Dv2, QLU-C10D and FACT-8D were 0.71 (0.19), 0.72 (0.24) and 0.58 (0.18), respectively. The strong ceiling effects were observed for the EQ-5D-5 L utility score. Additionally, strong ceiling effects were observed for all of the five dimensions of the EQ-5D-5 L, ranging from 40.8% (AD) to 91.6% (SC). For the SF-6Dv2, ceiling effects were observed for five of the six dimensions ranging from 21.7% (Mental Health/MH) to 51.3% (Pain), except for vitality (9.4%). In addition, four (namely, physical functioning, role limitation, social functioning and vitality) of the six dimensions showed slight-to-moderate floor effects, ranging from 19 to 29%. Ceiling effects were observed for all of the dimensions of the two cancer-specific measures. In the QLU-C10D, most of the participants selected “no problem” for role limitation (65.4%), followed by “appetite” (62.4%) and “nausea” (65.2%). The strength of the ceiling effect was lower for the FACT-8D than for the QLU-C10D, with slightly more than half of the respondents reporting “no problem” for the support and pain dimensions of the FACT-8D. Approximately 18% of the participants reported “no problem” for the sleep dimension, which was the lowest percentage among all of the FACT-8D dimensions.

**Table 2:** Measurement profile for EQ-5D-5 L, SF-6Dv2, QLU-C10D, and FACT-8D

	Level 1		Level 2		Level 3		Level 4		Level 5		Level 6	
	n	%	n	%	n	%	n	%	n	%	n	%
EQ-5D-5L												
Mobility	453	84.8	62	11.6	13	2.4	2	0.4	4	0.7		
Self-care	489	91.6	32	6	6	1.1	-	-	7	1.3		
Usual activities	475	89	44	8.2	10	1.9	2	0.4	3	0.6		
Pain/discomfort	298	55.8	201	37.6	28	5.2	6	1.1	1	0.2		
Anxiety/depression	218	40.8	235	44	63	11.8	10	1.9	8	1.5		
SF-6Dv2												
Mental health	116	21.7	161	30.1	208	39	37	6.9	12	2.2		
Pain	274	51.3	121	22.7	104	19.5	26	4.9	6	1.1	3	0.6
Physical functioning	128	24	246	46.1	92	17.2	49	9.2	19	3.6		
Role limitation	131	24.5	133	24.9	188	35.2	58	10.9	24	4.5		
Social functioning	155	29	122	22.8	152	28.5	76	14.2	29	5.4		
Vitality	50	9.4	131	24.5	250	46.8	79	14.8	24	4.5		
QLU-C10D												
Appetite	333	62.4	154	28.8	30	5.6	17	3.2				
Bowel	278	52.1	206	38.6	40	7.5	10	1.9				
Emotional functioning	176	33	254	47.6	64	12	40	7.5				
Fatigue	130	24.3	286	53.6	69	12.9	49	9.2				
Nausea	348	65.2	146	27.3	24	4.5	16	3				
Pain	313	58.6	189	35.4	24	4.5	8	1.5				
Physical functioning	210	39.3	192	36	98	18.4	34	6.4				
Role limitation	349	65.4	137	25.7	27	5.1	21	3.9				
Sleep	240	44.9	227	42.5	38	7.1	29	5.4				
Social functioning	134	25.1	282	52.8	65	12.2	53	9.9				
FACT-8D												
Fatigue	163	30.5	181	33.9	126	23.6	35	6.6	29	5.4		
Nausea	306	57.3	112	21	80	15	22	4.1	14	2.6		
Pain	269	50.4	166	31.1	79	14.8	11	2.1	9	1.7		
Sad	181	33.9	169	31.6	119	22.3	37	6.9	28	5.2		
Sleep	96	18	151	28.3	171	32	67	12.5	49	9.2		
Support	270	50.6	139	26	85	15.9	34	6.4	6	1.1		
Work	138	25.8	119	22.3	127	23.8	76	14.2	74	13.9		
Worry	114	21.3	190	35.6	123	23	45	8.4	62	11.6		

**Table 3:** Measurement profile at scale level

	Mean	Standard deviation	Median	Range	Ceiling effects %	Floor effects %
EQ-5D-5 L						
EQ-VAS	78.4	17.4	80.5	0–100	10.1	0.4
Utility score	0.89	0.16	0.95	−0.37–1	32.3	0.2
Full health (11111) [%]	33.0					
SF-6D						
Utility score	0.71	0.19	0.73	−0.24–1	5.6	0.2
Full health (11111) [%]	5.6					
QLU-C10D						
Utility score	0.72	0.24	0.78	−0.19–1	9.2	0.2
Full health (11111) [%]	6.6					
FACT-8D						
Utility score	0.58	0.18	0.59	−0.34–1	0.2	0.2
Full health (11111) [%]	0.0					

**Fig. 1:**
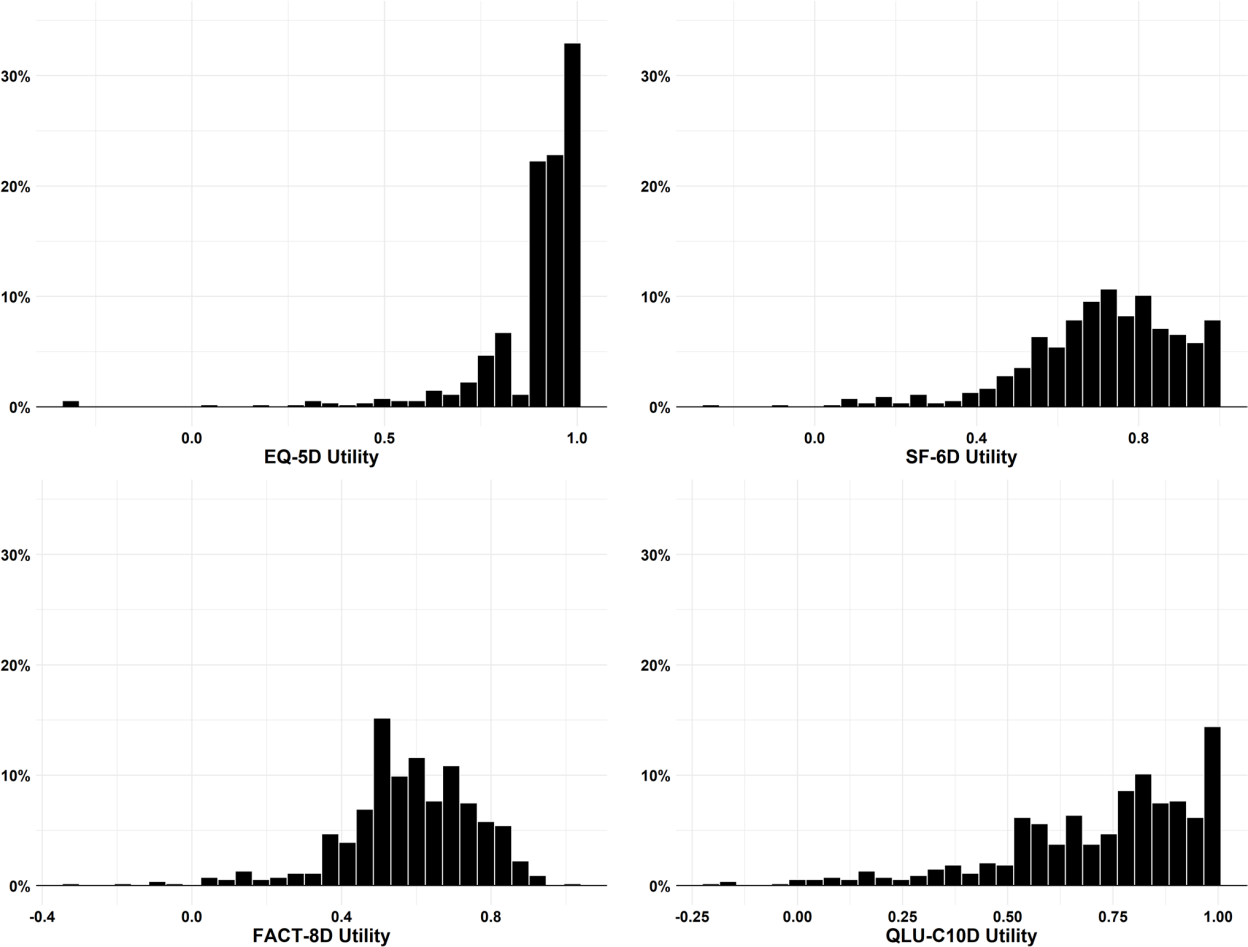
Score distribution of four measurements

The utility score distributions for the four measures are presented in Fig. 1. EQ-5D-5 L and QLU-C10D utility scores both showed strong negative skewness, with EQ-5D-5 L having a higher proportion of respondents reporting full health. SF-6D and FACT-8D score distributions, while not normal, displayed milder skewness than the other two measures.

### Convergent validity

**Fig. 2:**
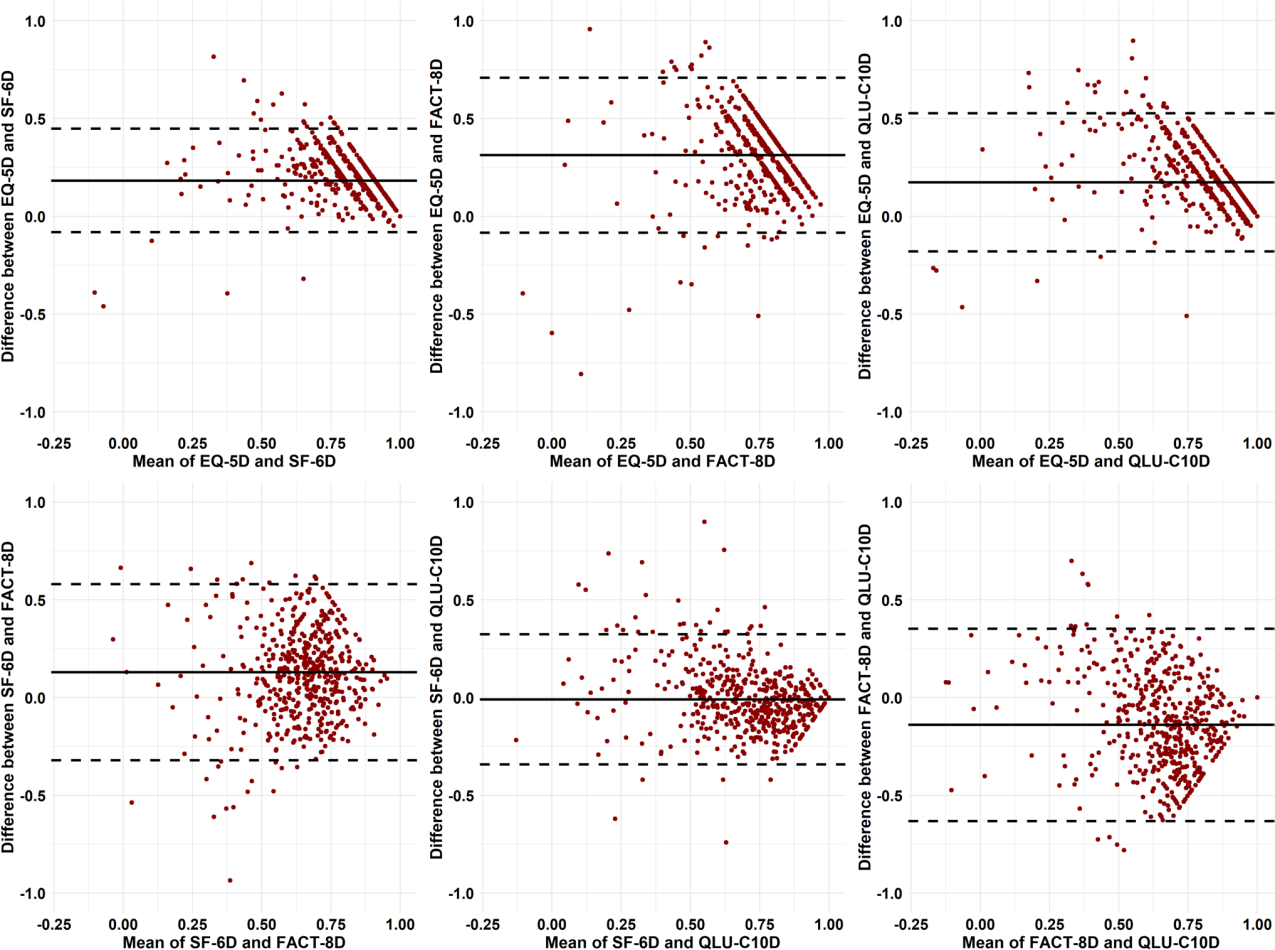
The B-A plot and distribution plot

The convergent validity of the four measures is presented in Table 4. We observed statistically significant correlations between all of the dimension pairs that were hypothesized to measure similar latent traits. Specifically, 15 pairs, which exhibited strong correlations (range, 0.5–0.77). The relationships of dimensions between QLU-C10D and FACT-8D were stronger than all other hypothesized pairs in this study. The correlation coefficients ranged from 0.41 (between role limitation in the QLU-C10D and work in the FACT-8D) to 0.77 (between nausea in both the QLU-C10D and FACT-8D). There are 14 pairs showed moderate correlations (*r* = 0.31–0.49), including seven pairs with EQ-5D-5 L dimensions (*r* = 0.31–0.45), six pairs with SF-6Dv2 dimensions (*r* = 0.41–0.47) and two pairs between the QLU-C10D and FACT-8D (*r* = 0.41 and 0.49, respectively). Moreover, the EQ-5D-5 L and SF-6Dv2 utility scores exhibited good agreement (ICC = 0.73). The SF-6Dv2 utility score displayed better agreement with the two cancer-specific measures than the EQ-5D utility score. However, the agreement between the QLU-C10D and FACT-8D utility scores was poor (*r* = 0.31). The Bland–Altman plots graphically indicated that the agreement was acceptable for all comparisons with a small mean difference, as few observations were outside the limits of agreement (Fig. 2).

**Table 4:** Correlations between hypothesized correlations between measures

No	Hypothesized correlations			Correlation coefficient
1	EQ-5D MO	~	SF-6D PF	0.44^***^
2	EQ-5D SC	~	SF-6D SF	0.29^***^
3	EQ-5D PD	~	SF-6D PA	0.37^***^
4	EQ-5D AD	~	SF-6D MH	0.7^***^
5	EQ-5D MO	~	QLU PF	0.42^***^
6	EQ-5D UA	~	QLU RL	0.44^***^
7	EQ-5D UA	~	QLU SF	0.31^***^
8	EQ-5D PD	~	QLU PA	0.58^***^
9	EQ-5D AD	~	QLU EF	0.61^***^
10	EQ-5D PD	~	FACT PA	0.6^***^
11	EQ-5D AD	~	FACT SA	0.55^***^
12	EQ-5D AD	~	FACT WR	0.45^***^
13	SF-6D SF	~	FACT WO	0.43^***^
14	SF-6D PA	~	FACT PA	0.68^***^
15	SF-6D MH	~	FACT SA	0.57^***^
16	SF-6D MH	~	FACT WR	0.43^***^
17	SF-6D VT	~	FACT FA	0.42^***^
18	SF-6D PF	~	QLU PF	0.47^***^
19	SF-6D RL	~	QLU RL	0.41^***^
20	SF-6D SF	~	QLU SF	0.49^***^
21	SF-6D PA	~	QLU PA	0.6^***^
22	SF-6D MH	~	QLU EF	0.64^***^
23	SF-6D VT	~	QLU FA	0.66^***^
24	QLU RL	~	FACT WO	0.41^***^
25	QLU EF	~	FACT SA	0.6^***^
26	QLU EF	~	FACT WR	0.49^***^
27	QLU PA	~	FACT PA	0.7^***^
28	QLU FA	~	FACT FA	0.5^***^
29	QLU SL	~	FACT SL	0.67^***^
30	QLU NA	~	FACT NA	0.77^***^

No	Hypothesized correlations			ICC value
31	EQ-5D utility	~	SF-6D utility	0.73^***^
32	EQ-5D utility	~	QLU-C10D utility	0.65^***^
33	EQ-5D utility	~	FACT-8D utility	0.31^***^
34	SF-6D utility	~	QLU-C10D utility	0.7^***^
35	SF-6D utility	~	FACT-8D utility	0.23^***^
36	QLU-C10D utility	~	FACT-8D utility	0.31^***^

*** p < 0.001

### Known-group validity

Table 5 presents the known-group validity data for the four measures. Overall, the generic measures (i.e., the EQ-5D-5 L and SF-6Dv2) showed higher sensitivity than the cancer-specific measures (i.e., the QLU-C10D and FACT-8D) in differentiating between the participants from the different groups of functioning ability, treatment and cancer stage. The EQ-5D-5 L was the only measure that could identify differences in HRQoL between all the subgroups. Although the SF-6Dv2, QLU-C10D and FACT-8D could distinguish participants diagnosed with cancer stages I or IIA as having a higher HRQoL than those diagnosed with cancer stage IIB or higher, the differences were not statistically significant. While the F-statistics demonstrated that the EQ-5D had a stronger discriminant power than the other measures in terms of self-care ability, use of assistive tools and cancer stage, the SF-6D was more discriminant in terms of caregiver and treatment status.

**Table 5:** Known-group validity

	*N*	EQ-5D utility	SF-6D utility	QLU-C10D utility	FACT-8D utility
Self-care ability					
Unable	49	0.22 (0.74)	0.15 (0.15)	0.25 (0.38)	0.18 (0.17)
Little	142	0.22 (0.43)	0.20 (0.17)	0.32 (0.23)	0.49 (0.2)
A lot	343	0.77 (0.18)	0.54 (0.19)	0.49 (0.27)	0.52 (0.23)
Fully capable		0.93 (0.09)	0.75 (0.16)	0.77 (0.19)	0.59 (0.17)
F-statistics		322.5	203.1	151.5	20.22
p-value		< 0.001	< 0.001	< 0.001	< 0.001
Using assistive tools					
Not at all	397	0.92 (0.11)	0.73 (0.17)	0.74 (0.16)	0.59 (0.17)
Rare	64	0.66 (0.21)	0.44 (0.23)	0.41 (0.21)	0.45 (0.26)
Sometimes	49	0.56 (0.42)	0.31 (0.32)	0.41 (0.11)	0.53 (0.18)
Always	24	0.32 (0.72)	0.44 (0.38)	0.44 (0.14)	0.4 (0.21)
F-statistics		192.2	82.82	49.59	12.28
p-value		< 0.001	< 0.001	< 0.001	< 0.001
With a caregiver					
No	256	0.93 (0.08)	0.77 (0.16)	0.78 (0.19)	0.6 (0.16)
Yes	278	0.86 (0.2)	0.66 (0.21)	0.66 (0.26)	0.56 (0.2)
F-statistics		23.13	42.72	38.08	5.69
p-value		< 0.001	< 0.001	< 0.001	0.02
Treatment status					
Treatment completed	281	0.92 (0.12)	0.77 (0.16)	0.79 (0.19)	0.6 (0.15)
Being treated	252	0.86 (0.19)	0.65 (0.21)	0.64 (0.26)	0.56 (0.21)
F-statistics		18.81	56.52	57.83	5.71
p-value		< 0.001	< 0.001	< 0.001	0.02
Cancer stage					
I and IIA (no spread)	197	0.92 (0.11)	0.73 (0.18)	0.59 (0.16)	0.74 (0.23)
≥ IIB (spread)	311	0.88 (0.19)	0.70 (0.20)	0.57 (0.19)	0.71 (0.24)
F-statistics		3.47	2.35	0.40	1.48
p-value		0.03	0.09	0.67	0.23

## Discussion

This study is the first to assess the measurement properties of two generic and two cancer-specific PBMs in a group of patients with CHL. Overall, the generic measures (i.e., the EQ-5D-5 L and SF-6Dv2) showed better measurement performance than the two cancer-specific measures (i.e., the QLU-C10D and FACT-8D). The EQ-5D-5 L demonstrated superior convergent and known-group validity to the SF-6Dv2, while the SF-6Dv2 exhibited milder ceiling and floor effects than the EQ-5D-5 L. These findings demonstrate that the generic PBMs are sensitive enough to measure HRQoL in survivors of CHL. However, it should be noted that more than half of our sample consisted of CHL patients currently undergoing treatment. Consequently, their quality of life, as measured by the cancer-specific measure, appeared lower than when assessed with the generic measure. This discrepancy arises because the cancer-specific measure might be more sensitive to domains that specifically affect cancer patients’ quality of life, resulting in more accurate outcomes.

We found that the average utility scores of the SF-6D, QLU-C10D and FACT-8D were lower than that of the EQ-5D-5 L and their ceiling effects (range, 9.4–65.4%) were weaker than those of the EQ-5D-5 L (range, 40.8–91.6%). Previous studies have provided mixed evidence among cancer patients. For instance, Gamper et al. demonstrated similar findings when comparing the QLU-C10D with the EQ-5D-3 L in patients from UK, Australia, and Italy [21]. Nahvijou et al. showed that the SF-6D generated a higher utility score than the EQ-5D-5 L in patients with breast cancer [22]. Kim et al. reported that the QLU-C10D generated a slightly higher utility score than the EQ-5D-3 L [22]. Pan et al. compared QLU-C1-D and EQ-5D-5 L among 243 cancer patients in China and they found that mixed results when comparing QLU-C10D and EQ-5D-5 L utility scores, largely depending on the choice of value sets [23]. Comparisons have been reported between the EQ-5D-5 L and FACT-8D is limited. Herdman et al. found that the EQ-5D-5 L performed as well or better than the FACT-8D in a multi-center RCT study involving 250 cancer patients [24]. There may be several explanations for these findings. For example, the EQ-5D-5 L has fewer dimensions than the SF-6D, QLU-C10D and FACT-8D, which may decrease its utility. Another reason is that the utilities of the two cancer-specific PBMs were developed based on discrete choice experiment (DCE) including duration, while most of the EQ-5D-5 L utilities were estimated based on a combination of time trade-off (TTO) and DCE techniques. The difference in valuation methods may result in systematic differences in utility. For instance, Xie et al. found that health utility scores derived from DCEs using the duration method were more likely to produce lower utility scores than the scores derived using both TTO and DCE [25].

The correlations between the two cancer-specific PBMs—the QLU-C10D and FACT-8D—were found to be stronger than those between the cancer-specific and generic PBMs. This is not surprising, as the two cancer-specific measures were developed to assess the health status specifically for patients with cancer, and their descriptive systems include the most important aspects of HRQoL for this population. For example, the correlation coefficient for nausea between the QLU-C10D and FACT-8D was the highest among all of the hypothesized pairs. However, we found that the association between the utility scores of these two measures was weak (ICC = 0.31). One possible explanation for this finding is that there is no Chinese value set for the FACT-8D, and the Australian preference weights that we used to calculate the utility score may not reflecting the preferences of the Chinese population. We further assessed the correlations between FACT-8D and the other three measures, using the Australian value sets to estimate the utility score. We found the ICC value increased, but not sufficiently. There is currently a lack of quantitative evidence for a direct comparison between the QLU-C10D and FACT-8D. A previous study revealed that the PITS state in the FACT-8D was significantly lower than in the QLU-C10D. This suggests that the PITS state depicted in the FACT-8D represents a more severe condition compared to the one in QLU-C10D, potentially resulting in a lower correlation between the two measures [26]. Another study that assessed the content validity of five PBMs in patients with cancer demonstrated that the FACT-8D had the best content validity in terms of relevancy among all of the evaluated measures, including the QLU-C10D [27]. In addition, we found a strong association between the utility scores of the EQ-5D and SF-6D, but the SF-6D dimensions showed stronger correlations with similar dimensions of the two cancer-specific PBMs than with the corresponding dimensions of the EQ-5D-5 L. Furthermore, the FACT-8D exhibited stronger correlations with the EQ-5D-5 L and SF-6D than with the QLU-C10D. A previous study provided qualitative evidence that the EQ-5D-5 L has good content validity in terms of comprehensibility [27], but there is a lack of evidence regarding the performance of the SF-6D compared with cancer-specific PBMs, and this requires further evaluation.

Furthermore, although the four instruments aim to measure dimensions of HRQoL, some constructs may vary. For instance, the item “sleep” in the QLQ-C30 is phrased negatively as “Have you had trouble sleeping?”, while in the FACT-G, it is presented positively as “I am sleeping well.” Such variations in phrasing can potentially affect the way participants interpret and respond to the question, thereby influencing the overall assessment of HRQoL. It may be a potential issue where instruments that measure ostensibly similar constructs could yield different results. This discrepancy could be due to subtle differences in the constructs themselves or variations in the precision and methodology of the measuring instruments [28]. Further exploration of the content validity of measures in this population is warranted.

Overall, the four PBMs we evaluated demonstrated good discriminant ability in differentiating HRQoL between at-risk groups, indicating satisfactory known-group validity. The FACT-8D performed less effectively than the other three PBMs in differentiating the cancer stage, which has been reported by a previous study [24]. A possible reason for the discrepancy could be the use of Australian preference weights to estimate the utility score for FACT-8D. This method may not fully capture the preferences of the Chinese population concerning the HRQoL of cancer. Furthermore, the Australian FACT-8D valuation study showed that identical coefficients were assigned to different severity levels in certain dimensions (e.g., fatigue, sleep, work, support, and worry). This could potentially limit its ability to differentiate between various levels of health issues [18]. The F-statistics confirmed that generic PBMs were more sensitive than cancer-specific PBMs in differentiating between patients. We found that while the utility scores of generic PBMs were more relevant for daily life functioning, such as self-care, use of assistive tools and caregivers, the utility score of the QLU-C10D was more relevant for treatment status. This finding contradicts the findings of a previous study by Gamper et al. [21], who found that a more comprehensive descriptive system gives the QLU-C10D a greater advantage compared to generic PBMs in distinguishing between clinically known groups. However, it is worth noting that Gamper et al. used an older sample with more than 80% of the participants reporting a cancer stage of 0 or I. In contrast, Pan et al. used a sample comparable to ours and found that the EQ-5D-5 L utilities generated higher F-statistic values than the corresponding QLU-C10D utilities [23]. Additionally, the variation in the EQ-5D-5 L utility score was less than that in the other three measures, which is possibly because it only includes five dimensions, resulting in a milder impact of disease on the overall score [23]. Furthermore, our known-group validity analysis revealed that the EQ-5D-5 L consistently showed the largest difference in the mean utility score between subgroups in three out of five comparisons. For instance, among patients who always used assistive tools, the mean utility score for the EQ-5D-5 L was 0.92, while among those who never used them, it was 0.32, resulting in a difference of 0.6. In comparison, the mean differences in the utility scores for the SF-6D, QLU-C10D and FACT-8D were 0.29, 0.3 and 0.19, respectively. This suggests that the utility gain in cost–utility analyses is likely to be larger for the EQ-5D-5 L than for the other measures.

The finding that generic preference-based measures exhibited better measurement properties than cancer-specific measures in CHL has significant implications. It suggests that generic measures can offer a reliable and valid assessment of quality of life in this patient population not worse than specific measures. This can facilitate broader applicability across different HL types, enabling more consistent comparisons and evaluations. For healthcare providers and policymakers, this reliability can aid in resource allocation and decision-making processes, ensuring that interventions are assessed on a standardized basis. Moreover, in the context of clinical trials and research, the superior measurement properties of generic measures imply that they could be prioritized for assessing patient outcomes, potentially simplifying the evaluation process without sacrificing accuracy. While cancer-specific measures are designed to capture the unique aspects of cancer patients’ experiences, the effectiveness of generic tools in this study suggests they are sufficiently sensitive to detect relevant changes in quality of life. This balance between specificity and generalizability can enhance the efficiency of both clinical practice and research, ultimately contributing to more patient-centered care and streamlined health economic evaluations in CHL.

### Limitations

Several limitations should be considered when interpreting the study findings. First, the sample was recruited from a volunteer pool through the internal network of a patient organization. These volunteers might be patients who are healthier than the average CHL survivors and frequent internet and social media users. This could potentially introduce a selection bias. Second, although online surveys are commonly used in this type of research, the data quality may not be entirely guaranteed because of the Internet-based format. Survivors of CHL may not have fully engaged in a long survey because of their poor physical and mental health, which may have affected the reliability of our findings. Finally, considering that we collaborated with a patient organization to collect data, medical information, such as comorbidities and clinical symptoms, were not collected from patients’ records, which may have affected the validity of our findings, especially known-group validity assessment.

## Conclusions

In conclusion, we found that all four of the PBMs demonstrated acceptable measurement properties in survivors of CHL. Overall, the EQ-5D-5 L performed better than the SF-6Dv2 in terms of its agreement with the cancer-specific PBMs and its discriminant ability. However, the SF-6Dv2 showed stronger associations with similar dimensions of the QLU-C10D and FACT-8D, indicating its high convergent validity. The utility score of the FACT-8D was lower than that of the other measures, suggesting the need for the future development of a Chinese value set for this measure. In addition, more evidence is urgently needed regarding the performance of the FACT-8D and SF-6Dv2 in patients with CHL. These findings have implications for selecting PBMs to estimate quality-adjusted life years to support economic evaluations for patients with CHL.

## Data Availability

Derived data supporting the findings of this study are available from the corresponding author on request.
